# Development and Validation of Risk Prediction Model for In-hospital Mortality Among Patients Hospitalized With Acute Exacerbation Chronic Obstructive Pulmonary Disease Between 2015 and 2019

**DOI:** 10.3389/fmed.2021.630870

**Published:** 2021-04-06

**Authors:** Fen Dong, Xiaoxia Ren, Ke Huang, Yanyan Wang, Jianjun Jiao, Ting Yang

**Affiliations:** ^1^Institute of Clinical Medical Sciences, China–Japan Friendship Hospital, Beijing, China; ^2^Department of Pulmonary and Critical Care Medicine, China-Japan Friendship Hospital, Beijing, China; ^3^National Clinical Research Center for Respiratory Diseases, Beijing, China; ^4^Institute of Respiratory Medicine, Chinese Academy of Medical Science, Beijing, China; ^5^Department of Medical Records, China–Japan Friendship Hospital, Beijing, China; ^6^Department of Medical Administration, China–Japan Friendship Hospital, Beijing, China

**Keywords:** prediction model, development, validation, in-hospital mortality, acute exacerbation of COPD

## Abstract

**Background:** In patients with chronic obstructive pulmonary disease (COPD), acute exacerbations affect patients' health and can lead to death. This study was aimed to develop a prediction model for in-hospital mortality in patients with acute exacerbations of COPD (AECOPD).

**Method:** A retrospective study was performed in patients hospitalized for AECOPD between 2015 and 2019. Patients admitted between 2015 and 2017 were included to develop model and individuals admitted in the following 2 years were included for external validation. We analyzed variables that were readily available in clinical practice. Given that death was a rare outcome in this study, we fitted Firth penalized logistic regression. C statistic and calibration plot quantified the model performance. Optimism-corrected C statistic and slope were estimated by bootstrapping. Accordingly, the prediction model was adjusted and then transformed into risk score.

**Result:** Between 2015 and 2017, 1,096 eligible patients were analyzed, with a mean age of 73 years and 67.8% male. The in-hospital mortality was 2.6%. Compared to survivors, non-survivors were older, more admitted from emergency, more frequently concomitant with respiratory failure, pneumothorax, hypoxic-hypercarbic encephalopathy, and had longer length of stay (LOS). Four variables were included into the final model: age, respiratory failure, pneumothorax, and LOS. In internal validation, C statistic was 0.9147, and the calibration slope was 1.0254. Their optimism-corrected values were 0.90887 and 0.9282, respectively, indicating satisfactory discrimination and calibration. When externally validated in 700 AECOPD patients during 2018 and 2019, the model demonstrated good discrimination with a C statistic of 0.8176. Calibration plot illustrated a varying discordance between predicted and observed mortality. It demonstrated good calibration in low-risk patients with predicted mortality rate ≤10% (*P* = 0.3253) but overestimated mortality in patients with predicted rate >10% (*P* < 0.0001). The risk score of 20 was regarded as a threshold with an optimal Youden index of 0.7154.

**Conclusion:** A simple prediction model for AECOPD in-hospital mortality has been developed and externally validated. Based on available data in clinical setting, the model could serve as an easily used instrument for clinical decision-making. Complications emerged as strong predictors, underscoring an important role of disease management in improving patients' prognoses during exacerbation episodes.

## Introduction

Chronic obstructive pulmonary disease (COPD) is a burdensome illness that accounts for 96% chronic respiratory disease deaths in China (1). In COPD progression, acute exacerbations can be easily triggered and affect patients' health. Severe exacerbations always increase the use of health service in hospital and cause great medical costs (2, 3). It may also lead to poor patient outcomes and even early death. Elderly patients are at particularly high risk of poor prognosis. Some co-existing morbidities, including COPD complications and comorbidities that occur during exacerbation episodes might worsen conditions and place patients at high risk of death. Prior studies have demonstrated an increased mortality risk in COPD patients with severe exacerbations (4).

Identifying high-risk patients for mortality during exacerbation episodes can help care providers deliver proper interventions to improve prognosis and avoid early death. Predicting health outcomes by the use of patient characteristics as predictors has been mentioned as a cornerstone in modern clinical medicine (5). An effective risk prediction model needs to incorporate prognostic factors that may influence patients' survival. Substantial efforts have been made to predict mortality in COPD patients, including the DECAF score, BAP-65, and 2008 scores for in-hospital mortality due to exacerbation (6). As health-care systems vary across nations, some predictors in the existing risk scores are not readily available in clinical setting. China has a heavy burden of COPD, with nearly 100 million COPD patients (7). Data routinely collected in clinical practice are seldom utilized for prognosis prediction in patients hospitalized for acute exacerbations of COPD (AECOPD). Studies on prediction models for in-hospital mortality in this subgroup of patients are limited.

Therefore, we developed and validated a risk prediction model for mortality in patients hospitalized with AECOPD. A clinical prediction tool was developed, incorporating easy-to-measure indicators that were readily available in clinical practice, aimed to help decision-making and optimize clinical care for patients in the real-world setting.

## Materials and Methods

### Study Patients

We performed a retrospective study in National Clinical Research Center for Respiratory Diseases, a 354-bed, medical and clinical research center in a tertiary hospital in Beijing, China. Eligible patients were individuals who were diagnosed as AECOPD, aged ≥40 years and admitted between January 1, 2015 and December 31, 2019. Patients with primary diagnosis of respiratory failure and secondary diagnosis of AECOPD were also included since their root cause of hospitalization was still AECOPD. All diagnoses in electronic medical records, including primary and secondary diagnoses, were determined by International Classification of Diseases, 10th Revision (ICD10) coding system. COPD was defined as J40-J44 in ICD-10 codes. In the study site, exacerbation was diagnosed when patients experienced at least any two of the following symptoms at clinics or emergency visits: dyspnea, increased sputum purulence and volume, increased cough and wheeze. To ascertain the accuracy of diagnoses, we reviewed medical records to ensure that our study patients were hospitalized due to acute exacerbations. Patients were excluded if they had been hospitalized within recent 30 days, or discharged against medical advice. Index admissions of eligible patients were included for analysis. The index admission was defined as the first admission for AECOPD during the study period.

### Potential Predictors and Outcome

We gathered the following information from electronic medical records: demographic characteristics (age, sex, marital status), concomitant diseases of COPD, source of admission (emergency or out-patient department), season and day of week of admission (weekday or weekend), and length of stay (LOS) at index admission. Concomitant diseases were determined by secondary diagnoses and classified into complications and comorbidities of COPD according to guidelines and researches (8–10). In this study, complications included respiratory failure, pulmonary heart diseases, hypoxemia, venous thromboembolism (VTE), pneumothorax, and hypoxic-hypercarbic encephalopathy. Comorbidities were cardio/cerebrovascular diseases, diabetes, respiratory infection, bronchiectasis, gastroesophageal reflux, obstructive sleep apnea syndrome, lung cancer, depression or anxiety, and osteoporosis. LOS was calculated as days by subtracting the admission date from discharge date. If patients were discharged and readmitted on the same day, their readmissions were actually the same hospitalization as the initial one. Their LOS were calculated as the summed days of hospital stay in each consecutive admission. If patients were admitted to the hospital for multiple times but not on the same day as aforementioned, we only analyzed their first admissions during 2015 and 2019. The primary outcome was all-cause mortality during hospitalization.

### Statistical Analysis

Data were summarized as number (percentage) for categorical variables and mean ± SD or median (interquartile ranges [IQRs]) for continuous variables where appropriate. *T*-test was adopted for normally distributed continuous variables and Mann–Whitney's test was for non-normally distributed ones. Categorical variables were analyzed using Chi's-square test or Fisher's exact test.

A large sample size can develop a robust model (11). We utilized all data on AECOPD admissions between 2015 and 2017 as a derivation dataset for model development and internal validation. Predictors that have plausible relationship with outcome are recommended as candidate predictors (12). Based on existing knowledge and literature review, we analyzed indicators that were potentially associated with death, including demographics, hospital admission and stay, season at admission, complications, and comorbidities of COPD. Baseline information were compared between patients alive and those who died. Univariable associations between potential predictors and mortality were examined. Indicators that were statistically significant were regarded as candidate predictors, including age, source of admission, LOS, respiratory failure, pneumothorax, and hypoxic-hypercarbic encephalopathy. To minimize potential overfitting, we anticipated a shrinkage factor of at least 0.9, and the shrinkage was 0.1 correspondingly. According to the new guideline on sample size for developing prediction model (11, 13), an estimated number of 510 participants were needed using the six aforementioned candidate predictor parameters and shrinkage factor. As death was a rare event in our study, penalized regression was adopted to avoid the overfitting arising from the low number of outcomes (14). We fitted penalized logistic regression for model selection using the LASSO method. Coefficients of predictors were estimated in Firth's penalized regression, which was an effective approach to overcome overfitting when the outcome was rare (14). A single-model analysis was also performed. The final model was determined by model selection and clinical relevance.

Performance of prediction model was evaluated by discrimination and calibration. C statistic, also known as the area under the curve (AUC), was a metric for the model's discrimination, which referred to the ability to classify patients who died from those alive in this study. Calibration characterized the accurate prediction of absolute death risk, being visually illustrated in a plot by comparing predicted and observed mortality rates at different levels. The curve's slope and intercept were estimated to quantify the calibration performance. Hosmer–Lemeshow test was utilized to examine discordances between the observed and predicted in-hospital mortality rate. In order to obtain a stable and unbiased estimate of discrimination and calibration, we deployed bootstrap to internally validate the performance of prediction model. The bootstrapped resample had the same size as derivation data. The modeling process was repeated in each resampled data. Optimism-corrected C statistic and calibration slope were estimated by bootstrapping 500 resamples according to Transport Reporting of a Multivariable Prediction Model for Individual Prognosis of Diagnosis (TRIPOD) statement (15).

To externally validate the prediction model, we longitudinally collected data on AECOPD patients hospitalized in the following 2 years (2018–2019). Discrimination and calibration of the prediction model were assessed by C statistics and calibration plot, respectively.

To apply this prediction model into clinical practice as a friendly used tool, we further transformed the model into a risk prediction algorithm and calculated risk score for individuals. According to beta coefficients of predictors in final prediction model and values of predictors, we assigned point for each predictor and calculated the total risk points (16). The threshold of risk score for in-hospital mortality was determined by the optimal Youden index. Also, a clinically relevant threshold was determined via visual inspection of an obvious increase in mortality risk. Sensitivity, specificity, and the Youden index were estimated at the thresholds.

This study was approved by China-Japan Friendship Hospital Clinical Research Ethics Committee (approval no. 2018-163-K119). Privacy and confidentiality of all patients' information were maintained. Patient informed consent was not required.

## Results

Totally, 1,096 AECOPD patients admitted between 2015 and 2017 were available for model development. The mean ± SD of age was 73 ± 10 years, and 67.8% were male. Most patients were admitted from outpatient department (55.1%) and at weekdays (89.8%). The majority of patients were admitted to respirology department, with only 3.84% admitted into intensive care unit (ICU). Cardio/cerebrovascular diseases were the predominant comorbidities (67.2%), and respiratory failure was the most common complication (26.1%). During hospitalization, 29 (2.6%) patients died. Compared to survivors, the non-survivors were older, more admitted from emergency, more frequently concomitant with respiratory failure, pneumothorax, hypoxic-hypercarbic encephalopathy, and had longer LOS (all *P* < 0.05) (Table 1). All these variables were associated with increased risk of mortality (Supplementary Table 1). Age, emergency admission, LOS, complications of respiratory failure, pneumothorax, and hypoxic-hypercarbic encephalopathy were determined as candidate predictors for model selection.

**Table 1:** Characteristics of COPD patients hospitalized for acute exacerbation between 2015 and 2017.

**Variables**		**All**	**Alive**	**Dead**	*P*-value^*^
		***N* = 1,096**	***N* = 1,067**	***N* = 29**	
Demographic characteristics	Age, years^**^	75 (66, 81)	75 (66, 81)	80 (72, 84)	0.0098
	Male	743 (67.8)	722 (67.7)	21 (72.4)	0.5893
	Married	1,048 (95.6)	1,021 (95.7)	27 (93.1)	0.5020
Admission and hospital stay	Admitted on weekend	112 (10.2)	108 (10.1)	4 (13.8)	0.5284
	Admitted from emergency	491 (44.9)	467 (43.9)	24 (82.8)	<0.0001
	Length of stay, days	11 (8, 15)	11 (8, 15)	22 (10, 31)	0.0002
Season at admission	Mar.–May	281 (25.6)	271 (25.4)	10 (34.5)	0.5387
	Jun.–Aug.	216 (19.7)	209 (19.6)	7 (24.1)	
	Sep.–Nov.	251 (22.9)	246 (23.1)	5 (17.2)	
	Dec.–Feb.	348 (31.8)	341 (32.0)	7 (24.1)	
Complications of COPD	Respiratory failure	286 (26.1)	259 (24.3)	27 (93.1)	<0.0001
	VTE	28 (2.6)	28 (2.6)	0 (0.0)	1.0000
	Pneumothorax	13 (1.2)	10 (0.9)	3 (10.3)	0.0040
	Pulmonary heart disease	225 (20.5)	218 (20.4)	7 (24.1)	0.6258
	Hypoxic-hypercarbic encephalopathy	27 (2.5)	23 (2.2)	4 (13.8)	0.0046
	Hypoxemia	31 (2.8)	31 (2.9)	0 (0.0)	1.0000
Comorbidities of COPD	Cardio/cerebrovascular Diseases	736 (67.2)	719 (67.4)	17 (58.6)	0.3214
	Bronchiectasis	93 (8.5)	90 (8.4)	3 (10.3)	0.7306
	Diabetes	183 (16.7)	181 (17.0)	2 (6.9)	0.2072
	Respiratory infection	137 (12.5)	133 (12.5)	4 (13.8)	0.7762
	Anxiety depression	17 (1.6)	17 (1.6)	0 (0.0)	1.0000
	Lung cancer	22 (2.0)	21 (2.0)	1 (3.4)	0.4489
	Osteoporosis	18 (1.6)	18 (1.7)	0 (0.0)	1.0000
	Reflux esophagitis	66 (6.0)	65 (6.1)	1 (3.4)	1.0000
	OSAS	19 (1.7)	19 (1.8)	0 (0.0)	1.0000

* *Chi-square or Fisher exact test was performed where appropriate. Age was abnormally distributed in all patients, survivors and non-survivors. They were expressed as median (interquartile range). Their mean ± SD were 73 ± 10, 73 ± 10, and 78 ± 8, respectively*.

### Model Development and Internal Validation

Using LASSO selection method, a model retaining age and LOS was selected based on schwarz bayesian criterion (SBC). Considering the aim to identify coexisting morbidities that may affect patients' outcome, we also incorporated another two significant predictors in single model analyses: respiratory failure and pneumothorax (Supplementary Table 2). Finally, age, LOS, respiratory failure, and pneumothorax were included into Firth's penalized logistic model for coefficient estimation. All the four predictors were statistically significant (all *P* < 0.05) (Table 2). The odds ratio (OR) and 95% confidence interval (CI) was 1.05 (1.002–1.09) for age, 27.47 (7.49–100.81) for respiratory failure, 6.71 (1.24–36.40) for pneumothorax and 1.03 (1.005–1.05) for LOS.

**Table 2:** Firth's penalized logistic regression for in-hospital mortality between 2015 and 2017.

**Variable**	**β coefficients**	***P*-value**	OR (95% CI)^*^
Intercept	−9.5187	<0.0001	–
Age, years	0.045	0.0382	1.05 (1.002, 1.09)
Respiratory failure	3.3131	<0.0001	27.47 (7.49, 100.81)
Pneumothorax	1.9041	0.0273	6.71 (1.24, 36.40)
Length of stay, days	0.0262	0.017	1.03 (1.005, 1.05)

* *OR, odds ratio; CI, confidence interval*.

**Figure 1:**
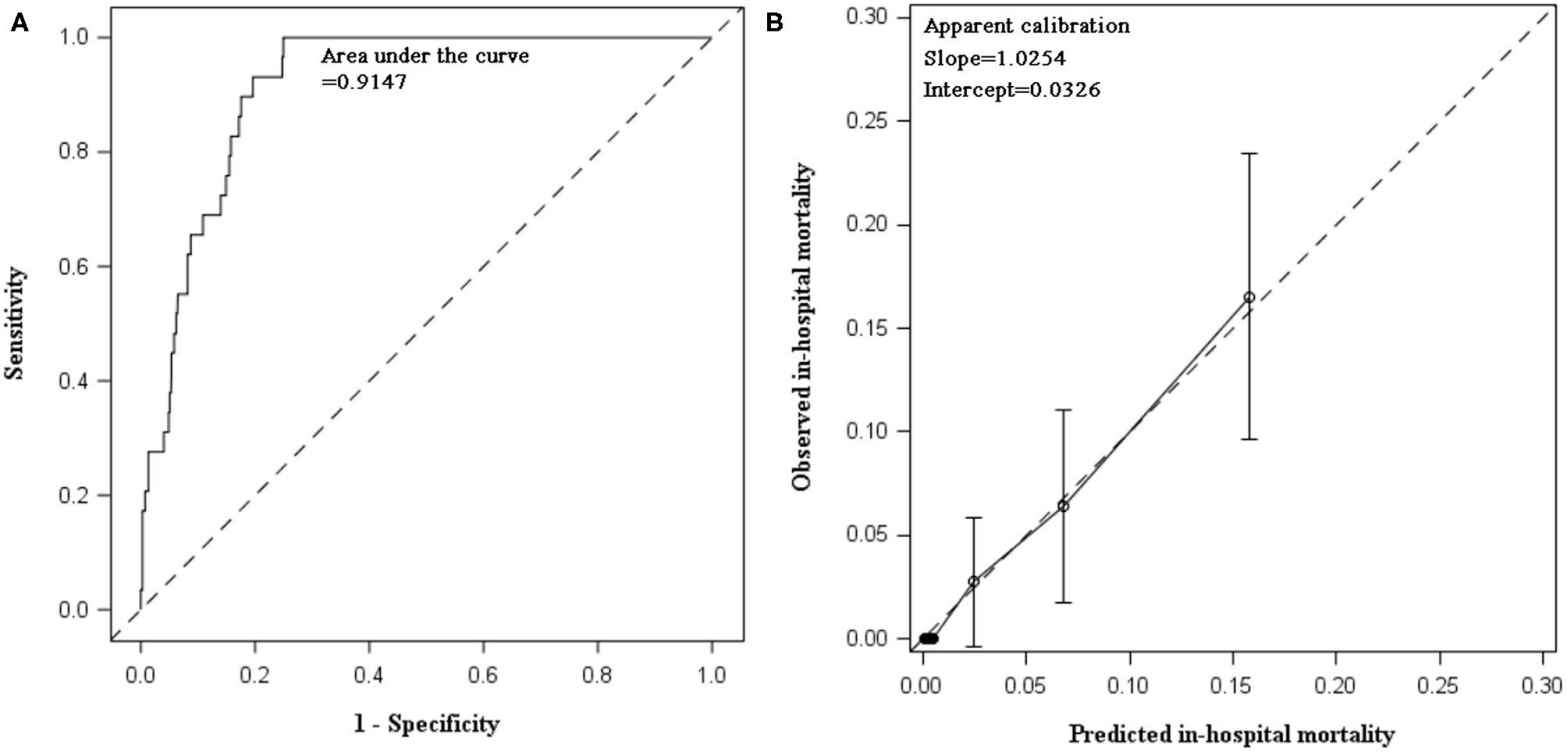
Discimination and calibration of in-hospital mortality prediction model in internal validation using data collected between 2015 and 2017. **(A)** AUC of prediabetes prediction model: the prediction model demonstrated excellent discrimination. The apparent C statistics and optimism-corrected C statistics were 0.9147 and 0.90887, respectively. After recalibration, C statistics remained the same and the estimate (95% CI) was still 0.9147 (0.8850, 0.9444). **(B)** Calibration plot of prediction model: calibration slope was 1.0254 (95% CI: 0.7276, 1.3233) and intercept was 0.0326 (95% CI: −0.7834, 0.8486). Hosmer–Lemeshow test was insignificant (chi-square statistics = 2.6249, df = 8, *P* = 0.9556).

In internal validation, C statistic of the prediction model was 0.9147 (95% CI 0.8850, 0.9444). After bootstrapping 500 times with replacement, the optimism was estimated as 0.00582. Optimism-corrected C statistic was 0.90887, indicating excellent discrimination of the prediction model to differentiate alive inpatients from those who died. The model had satisfactory apparent calibration with slope of 1.0254 and intercept of 0.0326. In calibration plot, the curve of observed vs. predicted mortality was closed to the diagonal. The Hosmer–Lemeshow test was insignificant (*P* = 0.9556). After adjustment for optimism, the slope was 0.9282 (Table 3, Figure 1).

**Table 3:** Apparent and validation performance of prediction model.

**Performance Statistics**	**Apparent performance in original sample**	**Internal validation**^**a**^			**External validation^**b**^**
		**Bootstrap performance**	**Test performance in original sample**	**Optimism corrected**	
C statistic	0.9147	0.91739	0.91157	0.90887	0.8176
Calibration^c^	Slope: 1.0254 Intercept: 0.0326	Slope: 1.0244Intercept: 0.0150	Slope: 0.9272 Intercept: −0.1874	Slope:0.9282	Slope: 0.5986 Intercept: −1.4804

a *Prediction model was internally validated in AECOPD patients admitted between 2015 and 2017. The estimates (95% confidence interval) of apparent performance of prediction model in original sample: 0.9147 (0.8850, 0.9444) for C statistic, 1.0254 (0.7276, 1.3233) for calibration slope and 0.0326 (−0.7834, 0.8486) for intercept. The apparent performance in the model derived from bootstrap sample was compared with the test performance obtained when applying the model to the original sample. Differences in apparent and test performances across all models were averaged to estimate the overall optimism for C statistics and slope. The optimism-corrected C statistics and slope were 0.90887 and 0.9282, respectively. The corrected slope (0.9282) was also shrinkage factor to adjust the original prediction model*.

b *External validation was performed in AECOPD patients admitted between 2018 and 2019. At external validation, the calibration slope reflects the combined effect of overfitting on the development data (2015–2017) and true differences in effects of predictors. Estimates (95% confidence interval) of prediction model performance were 0.8176 (0.7487, 0.8865) for C statistic, 0.5986 (0.2409, 0.9563) for calibration slope, and −1.4804 (−2.8037, −0.1571) for intercept, respectively*.

c *Intercept and slope of calibration plot were estimated in a logistic regression model with in-hosptial death events as outcome and linear predictor as the only independent variable*.

### External Validation

**Figure 2:**
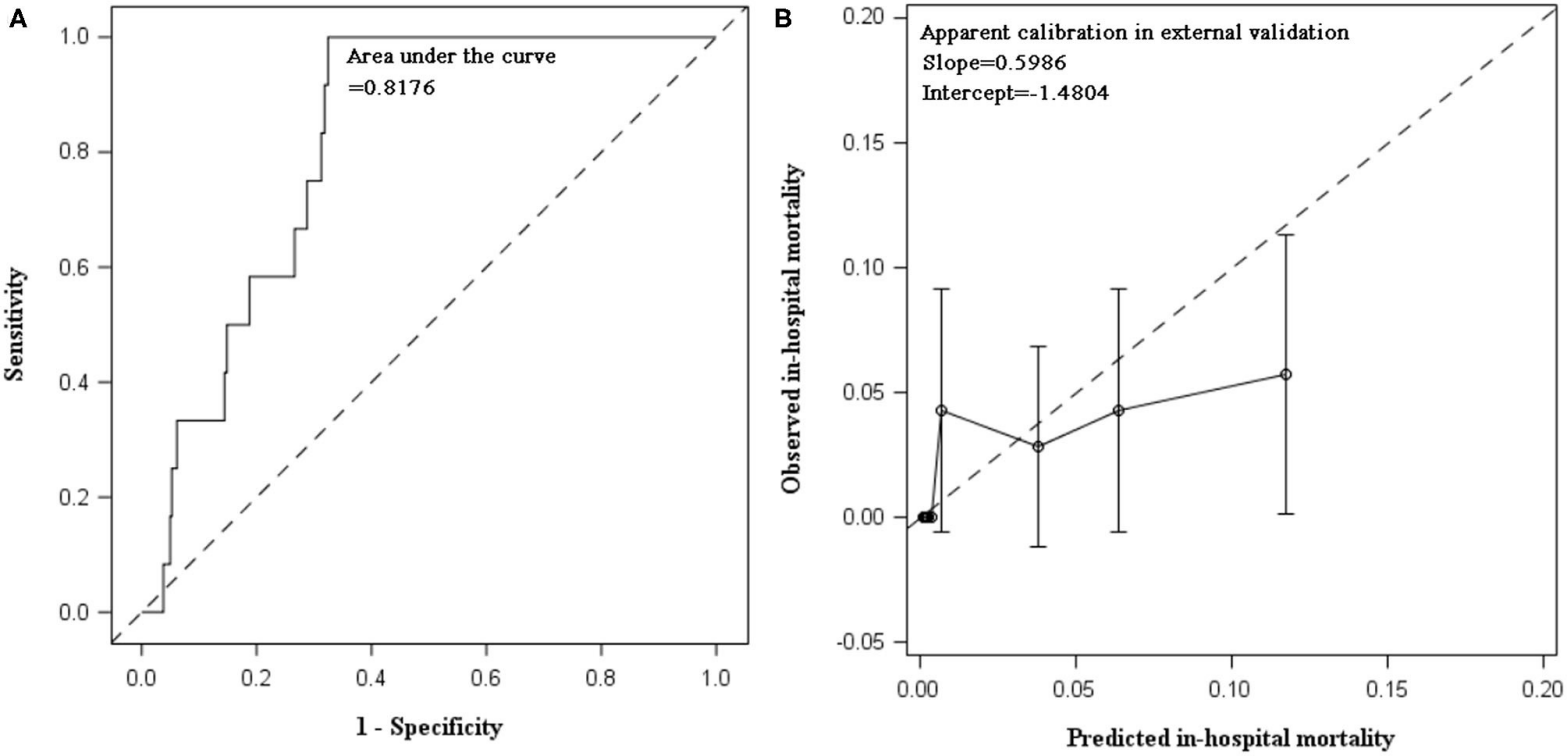
Discrimination and calibration of in-hospital mortality prediction model in external validation using data collected between 2018 and 2019. **(A)** AUC of in-hospital mortality prediction model: C statistic was 0.8176 (95% CI: 0.7487, 0.8865) in patients hospitalized for AECOPD during 2018 and 2019. **(B)** Calibration plot of prediction model: calibration slope was 0.5986 (95% CI: 0.2409, 0.9563) and intercept was −1.4804 (95% CI: −2.8037, −0.1571). Hosmer–Lemeshow test indicated poor calibration of our prediction model in AECOPD inpatients between 2018 and 2019. When stratified by predicted mortality, the prediction model was well-calibrated in low-risk patients with predicted mortality rate ≤10%. The Hosmer–Lemeshow test was insignificant (chi-square = 9.2052, *P* = 0.3253). Whereas, the death risk was overestimated in patients with predicted mortality rate greater than 10% (chi-square = 281.1386, *P* < 0.0001). The overall Hosmer–Lemeshow test result was significant (chi-square = 17.0856, *P* = 0.0292).

The external validation was performed in 700 AECOPD patients admitted in the following 2 years (2018 and 2019), of whom 12 (1.7%) patients died. Patients were mainly admitted to respirology department, with only 6.14% admitted into ICU. The mean ± SD of age was 72 ± 10 years and 73.1% were male. C statistics was 0.8176 (0.7487, 0.8865) for mortality prediction. In respect to calibration, we observed that the magnitude of difference between predicted and observed mortality varied along the death risk. The model was well-calibrated in low-risk patients with predicted mortality rate ≤10%, with a fairly large *P*-value in Hosmer–Lemeshow test (*P* = 0.3253). Whereas, mortality was overestimated in patients with predicted rate >10% (*P* < 0.0001). The overall Hosmer–Lemeshow test result was significant (*P* = 0.0292). The slope and intercept deviated from the ideal values of 1 and 0 (Table 3, Figure 2).

### Coefficient Adjustment and Intercept Recalibration

To account for optimism, predictor coefficients in original prediction model were adjusted using a shrinkage factor, which was the optimism-corrected slope of 0.9282 (Table 3). To maintain the overall apparent calibration, the intercept was re-estimated as −9.1935. Supplementary Table 3 demonstrated the final prediction model with shrunken predictor coefficients and recalibrated intercept. Applying the final model to derivation dataset of 2015–2017, there was no substantial change in C statistics since the ordering of predicted probabilities for models with original or shrunk coefficients are identical (17). But the model demonstrated excellent calibration in the derivation data of 2015–2017 with slope of 1.1048 and intercept of 0.43149 (Supplementary Figure 1A) and improved calibration with slope and intercept closer to 1 and 0 in the validation data of 2018–2019 (Supplementary Figure 1B).

### Risk Score and Stratification

**Figure 3:**
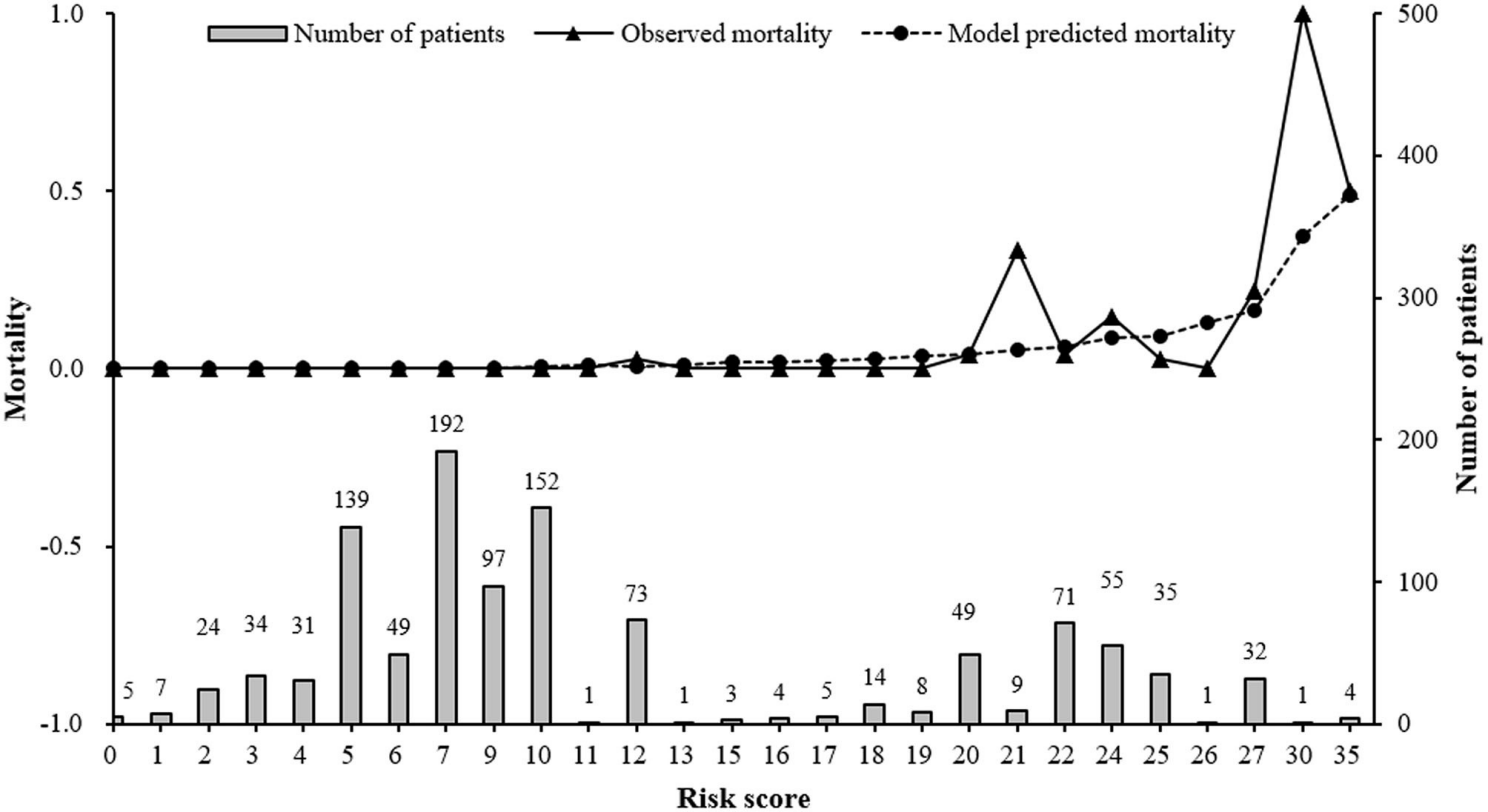
Observed, predicted in-hospital mortality along with risk score and distribution of risk score in AECOPD patients between 2015 and 2017. Model predicted mortality was calculated using our final prediction model: Log(mortality/(1-mortality)) = −9.1935 + 0.0418*age +3.0752*Respiratory failure + 1.7674*Pneumothorax + 0.0243*Length of stay Risk score was the total risk points, which was the sum of each predictor's risk point based on individual values.

After model adjustment with shrunken coefficients and recalibrated intercept, risk point of each predictor and total point were calculated on the basis of beta coefficients in the final model (Supplementary Table 4). The actual total risk points in AECOPD patients during 2015 and 2017 ranged from 0 to 35. The risk score of 20 was set as threshold to differentiate alive individuals from non-survivors, with an optimal Youden index of 0.7154, sensitivity 0.9310 (95% CI 0.8388–1.0000), and specificity 0.7844 (95% CI 0.7598–0.8091). The 20-risk score was also the point where both observed and predicted death risk started to increase, with almost all deaths (27/29) occurring in this subgroup of patients. The majority (76.55%) had <20 points (Figure 3). Predicted mortality at 20-risk score was 3.9%.

## Discussion

A simple risk prediction model was developed using routinely collected data in hospital. The recalibrated prediction model, incorporating demographic and clinical indicators, accurately predict in-hospital mortality in patients with AECOPD, with excellent discriminative and calibration ability in internal validation and good performance in external validation. Among patients experiencing acute exacerbation, advanced age, prolonged hospital stay, and concomitant morbidities were identified as important predictors for mortality. Respiratory failure and pneumothorax were particularly important concomitant morbidities, suggesting the key role of concomitant morbidities management in improvement of outcomes in AECOPD inpatients.

Several predictive models have been proposed for AECOPD mortality. The DECAF score is a well-known instrument for mortality prediction, with information gathered by medical and research staff following standard protocols (18). There exists a high heterogeneity of indicators used for model construction in the existing studies (6, 18, 19). Some indicators are not routinely collected in clinical practice, which might be attributed to disparities in health-care systems across nations. Also, definitions of outcome events and time frame of mortality varies. In a study performed in AECOPD patients visiting emergency, the outcome was short-term mortality, including mortality in hospital and within a week of discharge to home (19).

Consistent with previously reported studies on mortality in AECOPD, advanced age was recognized as a significant predictor for mortality in our study (18, 20). Respiratory failure emerged as a strong risk factor for in-hospital mortality, which is similar to a recent research on hypercapnia during respiratory failure increased the risk of mortality (21, 22). The increase in mortality risk was also observed in patients with respiratory failure in a cohort in Thailand (20). Respiratory failure is a serious complication in severe exacerbations. It reflects disease severity with a manifestation of dramatical drop in lung function. This condition can be modified through early interventions, for example, blood gas and proper management when admitted and utilization of ventilation or supplemental oxygen (20, 23, 24). Co-existing morbidities have been regarded as mortality predicting factors after hospital admission (4, 25, 26). But they were mostly measured as a comprehensive index such as Charlson. Analyses specific to a certain concomitant morbidity were not provided. To identify important prognostic factors for survival and guide clinical decision-making, it's necessary to specify concomitant morbidities that greatly affect the prognosis. Complications identified as mortality predictors in our study are potentially treatable and can be intervened early to curb disease progression, indicating significant clinical implications.

There are several limitations in this study. First, as the study was aimed to predict mortality risk in AECOPD patients, the study population was restricted to patients experiencing severe acute exacerbation that leads to hospital admission. Survival in patients with less severe exacerbations was not analyzed, which might compromise the generalizability of our prediction model. To strengthen the validity of our prediction model, it still needs to be validated in independent populations with diverse disease severity. Second, easy-to-measure indicators in daily clinical practice were used as candidate predictors. Differences in data collection in existing studies resulted in disparities in prognostic factors, making our findings incomparable with the prior ones (6, 18, 19). Third, this study focused on mortality risk during hospitalization, not including post-discharge deaths. It merits further study to address mortality prediction after hospital discharge. Finally, this study was performed in a single hospital center. Temporal validation in this center may limit the broad application of this risk score. There remains a need to validate it prospectively in an independent population from geographically different areas.

In spite of these weaknesses, this study sheds some light on clinical value of the routinely collected data in predicting patients' prognoses. The risk prediction model was built from four readily available indicators in clinical practice, including demographic and complications that commonly occur in AECOPD progression (e.g., respiratory failure and pneumothorax), which implies a straight-forward application of the prediction model in clinical practice. As data were collected in a standard process, the risk algorithm could be readily integrated into hospital information system, which may aid doctors in identifying high-risk individuals in an interactive and real-time way. This could facilitate implementation of tailored interventions to prevent fatal outcomes. Also, the model can be replicated in other hospitals owing to the standard process of data collection. Besides, the prediction model was simple with only four readily available predictors involved. It can save care providers' time in their busy work. Although the model was simple, it had good performance with high discriminative ability and good calibration. All available data were included for modeling by the use of bootstrap, and penalized regression was adopted to minimize potential overfitting arising from low mortality in this study. Our findings showed a shrinkage factor of >0.9, indicating no large overfitting (13). Furthermore, temporal validation was performed to assess external validity of the model, in which model was retrained and recalibrated, and any temporal trends that may affect the prediction could be captured (27).

## Conclusion

Complications emerged as important predictors for in-hospital mortality in acute exacerbated COPD patients requiring hospitalization. The model's prediction capability was satisfactory in terms of discriminating alive patients from individuals who died and estimating the absolute mortality risk in low-risk patients. This real-world data analysis indicates the potential value of risk algorithms as a toolkit to help doctors identify high-risk patients and adopt appropriate disease management to prevent death during COPD exacerbation.

## Data Availability Statement

The original contributions presented in the study are included in the article/Supplementary Material, further inquiries can be directed to the corresponding author/s.

## Ethics Statement

The studies involving human participants were reviewed and approved by China-Japan Friendship Hospital Clinical Research Ethics Committee (approval no. 2018-163-K119). Written informed consent for participation was not required for this study in accordance with the national legislation and the institutional requirements.

## Author Contributions

TY and JJ were involved in conceptualization, funding acquisition and supervision. FD and YW collected data. FD, XR, and KH analyzed and interpreted the data. FD wrote the draft of manuscript. TY, JJ, FD, XR, KH, and YW contributed to writing-review and approved the final manuscript. All authors contributed to the article and approved the submitted version.

## Conflict of Interest

The authors declare that the research was conducted in the absence of any commercial or financial relationships that could be construed as a potential conflict of interest.
