# Supplementary material for: Development and Validation of Risk Prediction Model for In-hospital Mortality Among Patients Hospitalized With Acute Exacerbation Chronic Obstructive Pulmonary Disease Between 2015 and 2019
Source: Front Med (Lausanne). 2021 Apr 6;8:630870. doi: 10.3389/fmed.2021.630870 (PMC8055833; doi:10.3389/fmed.2021.630870)
Supplement: Supplementary file 1 [file Data_Sheet_1.docx]

Supplementary Material

**Supplementary Table 1.** Univariable analysis for unadjusted association between potential predictors and in-hospital mortality

| **Variables** | | **OR 95% CI*** | **P value** |
| --- | --- | --- | --- |
| Demographic characteristics | Age, years | 1.05 (1.01, 1.10) | 0.0167****** |
|  | Male | 1.21 (0.54, 2.71) | 0.6434 |
|  | Married | 0.50 (0.13, 1.91) | 0.312 |
| Admission and hospital stay | Admitted on weekend | 1.56 (0.56, 4.35) | 0.395 |
|  | Admitted from emergency | 5.69 (2.24, 14.48) | 0.0003****** |
|  | Length of stay, days | 1.06 (1.04, 1.09) | <.0001****** |
| Season at admission | Mar.-May (ref) |  |  |
|  | Jun.-Aug. | 0.93 (0.36, 2.41) | 0.8742 |
|  | Sep.-Nov. | 0.58 (0.20, 1.65) | 0.3036 |
|  | Dec.-Feb. | 0.57 (0.22, 1.47) | 0.244 |
| Complications of COPD | Respiratory failured | 34.3 (9.31, 126.1) | <.0001****** |
|  | VTE | 0.62 (0.04, 10.89) | 0.7426 |
|  | Pneumothorax | 13.3 (3.58, 49.47) | 0.0001****** |
|  | Pulmonary heart disease | 1.30 (0.56, 3.01) | 0.5456 |
|  | Hypoxic-hypercarbic encephalopathy | 7.84 (2.61, 23.56) | 0.0002****** |
|  | Hypoxemia | 0.56 (0.03, 9.76) | 0.6892 |
| Comorbidities of COPD | Cardio/Cerebrovascular Diseases | 0.68 (0.32, 1.42) | 0.3021 |
|  | Bronchiectasis | 1.43 (0.46, 4.46) | 0.5416 |
|  | Diabetes | 0.44 (0.12, 1.64) | 0.2237 |
|  | Respiratory infection | 1.24 (0.44, 3.43) | 0.6852 |
|  | Anxiety depression | 1.02 (0.06, 18.78) | 0.9901 |
|  | Lung cancer | 2.56 (0.45, 14.49) | 0.2875 |
|  | Osteoporosis | 0.96 (0.05, 17.64) | 0.9782 |
|  | Reflux esophagitis | 0.81 (0.15, 4.29) | 0.7999 |
|  | OSAS | 0.91 (0.05, 16.62) | 0.9496 |

*OR=Odds ratio, CI=confidence interval** P<0.05

**Supplementary Table 2.** Single model analysis for associations between each block of potential predictors and in-hospital mortality

| **Block of potential predictors** | | **OR 95% CI*** | **P value** |
| --- | --- | --- | --- |
| Model 1:  Demographic characteristics | Age, years | 1.06 (1.01, 1.10) | 0.0112** |
|  | Male | 1.50 (0.67, 3.38) | 0.3221 |
|  | Married | 0.57 (0.15, 2.18) | 0.4154 |
| Model 2:  Admission and hospital stay | Admitted on weekend | 1.17 (0.41, 3.36) | 0.7678 |
|  | Admitted from emergency | 4.05 (1.55, 10.61) | 0.0044** |
|  | Length of stay, days | 1.05 (1.02, 1.07) | <0.0001** |
| Model 3:  Season at admission | Jun.-Aug. | 0.93 (0.36, 2.41) | 0.8742 |
|  | Sep.-Nov. | 0.58 (0.20, 1.65) | 0.3036 |
|  | Dec.-Feb. | 0.57 (0.22, 1.47) | 0.244 |
| Model 4:  Complications of COPD | Respiratory failure | 34.3 (9.63, 122.1) | <0.0001** |
|  | VTE | 0.34 (0.02, 6.32) | 0.4688 |
|  | Pneumothorax | 8.01 (1.63, 39.27) | 0.0103** |
|  | Pulmonary heart disease | 0.54 (0.23, 1.29) | 0.1663 |
|  | Hypoxic-hypercarbicencephalopathy | 2.44 (0.79, 7.54) | 0.1207 |
|  | Hypoxemia | 2.24 (0.08, 58.95) | 0.6299 |
| Model 5:  Comorbidities of COPD | Cardio/Cerebrovascular Diseases | 0.72 (0.35, 1.46) | 0.3568 |
|  | Bronchiectasis | 1.40 (0.46, 4.24) | 0.5523 |
|  | Diabetes | 0.48 (0.14, 1.64) | 0.239 |
|  | Respiratory infection | 1.20 (0.45, 3.19) | 0.7178 |
|  | Anxiety depression | 1.11 (0.07, 18.46) | 0.9436 |
|  | Lung cancer | 2.19 (0.39, 12.28) | 0.3745 |
|  | Osteoporosis | 0.80 (0.05, 13.56) | 0.8789 |
|  | Reflux esophagitis | 0.88 (0.18, 4.24) | 0.8741 |
|  | OSAS | 0.90 (0.05, 15.34) | 0.9426 |

*OR=Odds ratio, CI=confidence interval; ** P<0.05

**Supplementary Table 3.** Recalibration of prediction model for in-hospital mortality between 2015-2017

| **Variable** | **β coefficients in original model** | **Shrinkage factor** | **Shrunken predictor coefficients ^a^ and recalculated intercept** |
| --- | --- | --- | --- |
| Intercept | -9.5187 | - | -9.1935 ^b^ |
| Age, years | 0.045 | 0.9282 | 0.0418 |
| Respiratory failure | 3.3131 | 0.9282 | 3.0752 |
| Pneumothorax | 1.9041 | 0.9282 | 1.7674 |
| Length of stay, days | 0.0262 | 0.9282 | 0.0243 |

^a^ Predictor coefficients were shrunken by multiplying shrinkage factor with original coefficients of predictors in the original model.

^b^ To maintain the overall apparent calibration, intercept was recalculated by substracting the multiplications of shrunken coefficients with predictor values from the original linear prediction. Recalculated intercept was mean difference between original linear prediction and the summed multiplications of shrunken coefficients and predictor values.


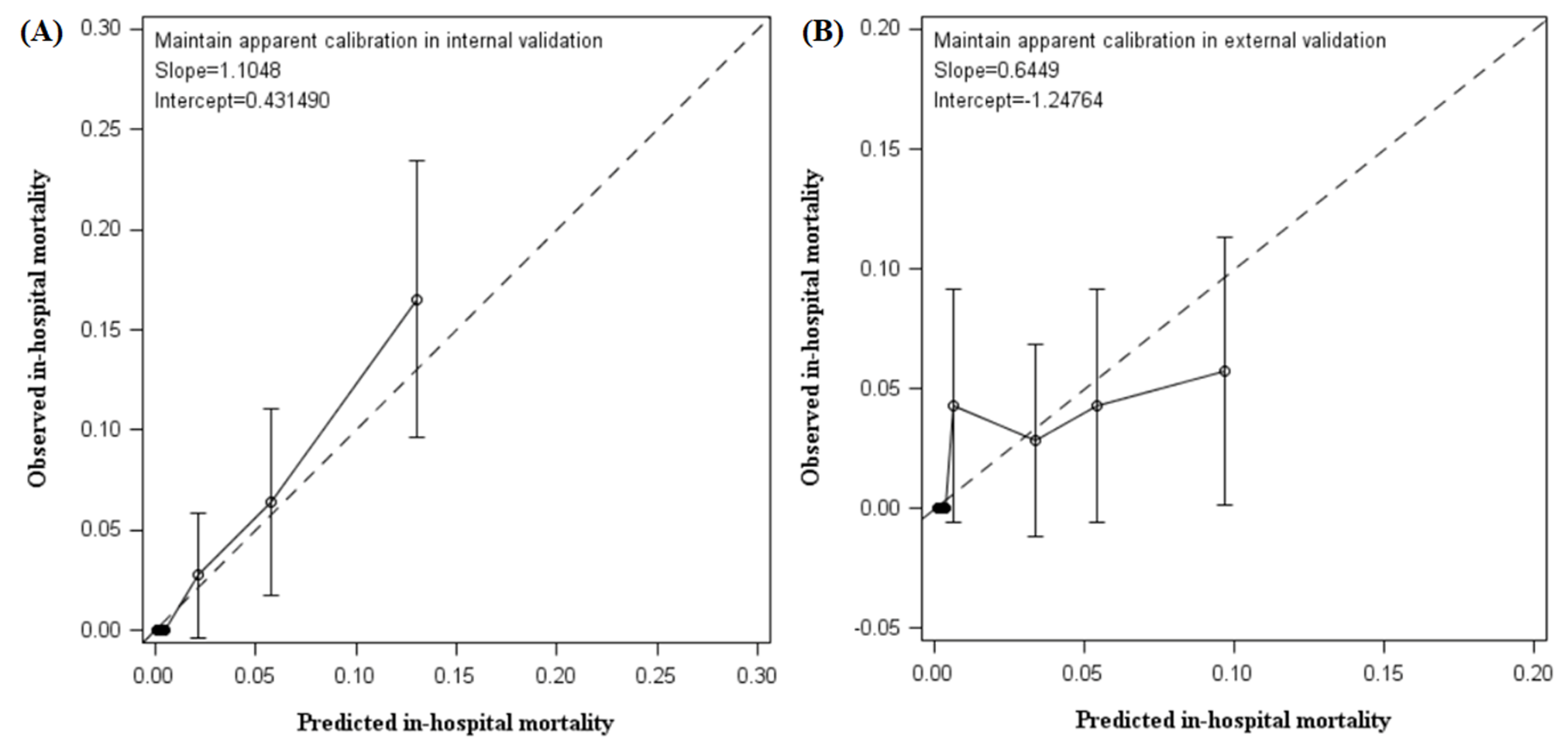


**Supplementary Figure 1.**Calibration plot of prediction model with recalibrated intercept and shrunken predictors:

A: internal validation in AECOPD patients hospitalized between 2015 and 2017, using prediction model with adjusted intercept -9.1935 and shrunken coefficient by 0.9282. Hosmer-Lemeshow test: Chi-square=3.773, P=0.8770

B: external validation in AECOPD patients hospitalized between 2018 and 2019 using prediction model with adjusted intercept -9.1935 and shrunken coefficient by 0.9282.The slope and intercept were closer to 1 and 0 compared to the original model, indicating improvement in calibration. The Hosmer and Lemeshow test was marginally statistically significant (Chi-square=16.3833, P=0.0372).

**Supplementary Table 4.** Risk points of predictors for in-hospital mortality

| **Predictor** | **Categories** | **Reference value** | **Base reference** | **Estimates of beta coefficients** | **Risk points** |
| --- | --- | --- | --- | --- | --- |
| Intercept |  | . | . | -9.1935 | . |
| Age, years | 40-49 | 44.5 | 44.5 | 0.0418 | 0 |
|  | 50-59 | 54.5 | 44.5 | 0.0418 | 2 |
|  | 60-69 | 64.5 | 44.5 | 0.0418 | 4 |
|  | 70-79 | 74.5 | 44.5 | 0.0418 | 6 |
|  | 80-97 | 88.5 | 44.5 | 0.0418 | 9 |
| Respiratory failure | 0 | 0 | 0 | 3.0752 | 0 |
|  | 1 | 1 | 0 | 3.0752 | 15 |
| Pneumothorax | 0 | 0 | 0 | 1.7674 | 0 |
|  | 1 | 1 | 0 | 1.7674 | 8 |
| Length of stay, days | 1-7 | 4 | 4 | 0.0243 | 0 |
|  | 8-14 | 11 | 4 | 0.0243 | 1 |
|  | ≥15 | 33 | 4 | 0.0243 | 3 |
